# Levobupivacaine Induces Ferroptosis by miR-489-3p/SLC7A11 Signaling in Gastric Cancer

**DOI:** 10.3389/fphar.2021.681338

**Published:** 2021-06-09

**Authors:** Shun-Hong Mao, Chun-Hua Zhu, Yu Nie, Jian Yu, Lei Wang

**Affiliations:** Department of Anesthesia, Cangzhou Central Hospital, Cangzhou, China

**Keywords:** gastric cancer, levobupivacaine, ferroptosis, mir-489-3p, SLC7A11

## Abstract

Gastric cancer is one of the most the prevalent malignancies and the therapeutic strategies for patients with gastric cancer remains limited. Local anesthetic levobupivacaine has demonstrated potential anti-cancer property, but its correlation with gastric cancer and ferroptosis is poor understood. Here, we identified the novel function of levobupivacaine in regulating ferroptosis of gastric cancer cells. The treatment of levobupivacaine suppressed gastric cancer cell viabilities and Edu-positive cell proportions. The gastric cancer cell growth was reduced by levobupivacaine *in vivo*. Moreover, the treatment of levobupivacaine enhanced erastin-induced inhibitory impact on gastric cancer cell viabilities. The levels of Fe^2+^/iron and lipid ROS were induced by levobupivacaine in erastin and RSL3-stimulated gastric cancer cells. levobupivacaine-upregulated miR-489-3p enhanced ferroptosis of gastric cancer cells by targeting SLC7A11. MiR-489-3p was involved in levobupivacaine-induced ferroptosis of gastric cancer cells. Levobupivacaine/miR-489-3p/SLC7A11 axis attenuates gastric cancer cell proliferation *in vitro*. Therefore, we concluded that the local anesthetic levobupivacaine induced ferroptosis of gastric cancer cells to repress gastric cancer cell growth by miR-489-3p/SLC7A11 axis.

## Introduction

Gastric cancer is a lethal disease that ranks the fifth most prevalent cancer worldwide, often diagnosed at advanced stage (Smyth et al., 2020). Ferroptosis is a newly discovered form of nonapoptotic cell death, characterized by iron-associated accumulation of lipid peroxides (lipid-ROS) (Dixon et al., 2012). An increasing number of researches have demonstrated the involvement of ferroptosis in tumor development and drug-resistance of multiple cancer types, including gastric cancer (Yagoda et al., 2007; Alvarez et al., 2017; Zhang et al., 2020). Therefore, targeting ferroptosis may be an efficient therapeutic strategy for gastric cancer.

Local anesthetics such as bupivacaine, levobupivacaine, and lidocaine, are indicated to be capable of affecting the progression of multiple cancers including gastric cancer, breast cancer and so on (Dan et al., 2018; Li et al., 2018). Levobupivacaine could notably inhibit the viability, migration, cell cycle of breast cancer MCF7 and MDA-MB-231 cells, also exhibited no cytotoxicity to non-cancerous MCF10A breast cell line (Li et al., 2018). Castelli and colleagues recently reported that treatment with levobupivacaine led to the counteracted proliferation and migration of melanoma cells (Castelli et al., 2020). Nevertheless, the function of levobupivacaine in gastric cancer is still unclear.

MicroRNAs (miRNAs) are a class of endogenous non-coding RNA, with highly conserved sequence of around 20 nucleotides in length (Lee and Dutta, 2009). The miRNAs usually function at post-transcriptional level, through targeting the 3′UTR region of target mRNAs and impede their expression (Lee and Dutta, 2009). Over the past decades, increasing evidences have presented the important role of miRNAs in development and progression of malignant cancers (Rupaimoole and Slack, 2017). For example, miR-149 functions in a context-dependent manner, either as a tumor suppressor or “onco-miRNA” in tumorigenesis (Ow et al., 2018). MiR-489-3p potentially regulate proliferation and glycolysis in melanoma (Yang et al., 2020).

SLC7A11 is a critical factor of the cystine-glutamate antiporter, and widely participates in the multiple biological processes of cancer development (Liu et al., 2019). Among the various functions of SLC7A11, Recent years, involvement of SLC7A11 in metabolism and ferroptosis of cancer cells has gained great attention (Fang et al., 2020; Hu et al., 2020). For example, it was reported that SLC7A11 overexpression promotes tumor growth partly through suppressing ferroptosis at a transcriptional level (Koppula et al., 2020). Lang ex al. proposed that repression of SLC7A11 facilitated the radiotherapy and immunotherapy of tumors through regulating ferroptosis (Lang et al., 2019).

In this work, we discovered that local anesthetic levobupivacaine induced ferroptosis in gastric cancer, suppressed cell proliferation both *in vitro* and *in vivo*. In addition, levobupivacaine treatment elevated the level of miR-489-3p, which further functioned as a sponge of SLC7A11 to suppress its expression. Overall, this work disclosed a potential therapeutic function of local anesthetic levobupivacaine in gastric cancer, and provided the possible mechanisms of miR-489-3p/SLC7A11 involved in this process.

## Materials and Methods

### Cell Culture and Reagents

Normal gastric epithelial GES-1 cell lines and HGC27 and SGC7901 gastric cancer cell line was obtained from the ATCC (namely the American Type Culture Collection), and maintained in the RPMI1640 medium (Thermo, United States) added with 10% Fasting Blood Sugar (FBS) (Gibco, United States) plus 1% penicillin/streptomycin (Solarbio, China). All cells were kept in an environment of 5% CO_2_ and temperature of 37°C. The levobupivacaine was purchased from Sigma. The dose of levobupivacaine was used at 2 mM according to the previous report (Kwakye et al., 2020).

### MTT

HGC27 and SGC7901 cells in a logarithmic phase were seeded into a 96-well plate at a density of 3 × 10^3^ cells/well. After 12-h incubation, the medium was replaced by fresh medium added with levobupivacaine. At the end time point (24, 48, and 72 h), 10 μl of 5 g/L MTT reagent (Thermo, United States) was added to each well for a continued 4-h culture in the incubator. Then the plate was emptied and added with 150 μl of Dimethyl Sulfoxide (DMSO) into each well. The values at OD 490 nm were detected through a microplate reader (PerkinElmer).

### 5-Ethynyl-2′-Deoxyuridine Assay

The proliferation of HGC27 and SGC7901 cells were detected by using a EdU assay kit (Beyotime) according to manufacturer’s protocol. In brief, cells were stained by diluted EdU solution for 2 h, washed and fixed by 4% paraformaldehyde. Next, cells were incubated with Hoechst33342 for 30 min in dark, washed and observed by using a fluorescence microscope (CarlZeiss, United States).

### Flow Cytometry

The apoptosis of HGC27 and SGC7901 cells was determined by a FITC-AnnexinV/PI apoptosis detection kit (Beyotime, China) following the manufacturer’s instruction. In brief, cells subjected to indicated treatment were collected in binding buffer, dual stained by FITC-AnnexinV and PI solution for 20 min. The samples were immediately analyzed by a C6 Flow cytometry (BD Biosciences).

### Xenograft Tumor Model

All animal experiments were conducted under the approval of Cangzhou Central Hospital. Ten SCID nude mice aged 6–8 weeks were purchased from Vital River Laboratory Animal Technology Co., Ltd. (Beijing, China), and subcutaneously injected with SGC7901 cells (5 × 106 cells per mouse) in left back. One week after feeding, the mice were randomly divided into two groups, the control and treatment group. For the next 25 days, the mice in treatment group were injected with erastin (15 mg/kg intraperitoneally) or co-treated with 40 μmol/kg body weight of levobupivacaine once a day. The body weight and tumor size were measured every 3 days. The levels of SLC7A11 (Abcam, United States) was detected by immunohistochemistry (IHC) staining in the mice. Animal care and method procedure were authorized by the Animal Ethics Committee of Cangzhou Central Hospital.

### Detection of Ferroptosis Biomarkers

Elevated iron level and accumulated lipid ROS were representative characteristics of ferroptosis. We used an iron assay kit (Beyotime) to examine the level of intracellular Fe^2+^. Briefly, the cells were homogenized to collect the supernatant, incubated with iron reducer, followed by labeling by iron probe. The OD 590 nm were detected in a microplate reader (PerkinElmer). For detection of lipid ROS, cells were stained with BODIPY C-11 dye (Beyotime) for 30 min, and subsequently detected by Flow cytometry (BD bioscience).

### Cell Transfection

HGC27 and SGC7901 cells at logarithmic growth phase were digested and placed in six-well plates at a density of 5 × 105 cells per well. The SLC7A11 overexpression plasmid was synthesized and purchased (Genscript, China), in which the CDS region of SLC7A11 was inserted into pcmv-3tag-1a. The siRNAs of SLC7A11 was purchased (Ribobio, China). The SLC7A11 siRNA targeted sequence: 5′-CCA​UUA​UCA​UUG​GCA​CCA​U-3′. miR-489-3p mimics, inhibitor, and the negative control (NC) were transfected by using the RFect siRNA/miRNA Transfection Reagent (Biogen, United States) following the manufacturer’s protocol, and placed in incubator for 24 h.

### RNA Extraction and Quantification

The quantification of miR-489-3p and SLC7A11 mRNA was performed by real-time fluorescence quantitative PCR (RT-PCR). The cells treated with indicated reagents were lyzed by Trizol (Life Technologies, United States) to obtain total RNAs. A total of 1 μg RNA was subjected to reverse transcription using a reagent kit (Thermo). The conversion of miRNA to cDNA was achieved *via* a SYBR RT-PCR kit (Takara, China). The fold change of RNAs was calculated by a e 2^−ΔΔCt^ method. The primers were as follows: GAPDH, sense, 5′-CTT​TGG​TAT​CGT​GGA​AGG​ACT​C-3′; anti-sense, 5′-GTA​GAG​GCA​GGG​ATG​ATG​TTC​T-3′; U6, sense, 5′-GCT​TCG​GCA​GCA​CAT​ATA​CTA​AAA​T-3′; anti-sense, 5′-CGC​TTC​ACG​AAT​TTG​CGT​GTC​AT-3′; SLC7A11, sense, 5′- GCT​GTG​ATA​TCC​CTG​GCA​TT -3′; anti-sense, 5′-GGC​GTC​TTT​AAA​GTT​CTG​CG-3′; miR-489-3p, sense, 5′- ACA​CTC​CAG​CTG​GGG​TGA​CAT​CAC​ATA -3′; anti-sense, 5′- TGGTGTCGTGGAGTCG -3′.

### Luciferase

The potential interaction between SLC7A11 and miR-489-3p was predicted on Targetscan website (http://www.targetscan.org/vert_71/). We constructed two luciferase reporter plasmids, the pmirGLO-SLC7A11-WT (WT) and pmirGLO-SLC7A11-Mut (Mut). The last 1,000 bp of the SLC7A11 3′UTR containing the miR-489-3p binding site was cloned in pmirGLO vector. To obtain SLC7A11-WT, the wild type sequence of the 3′UTR of SLC7A11 (wild type last 1,000 bp containing the miR-489-3p binding site: uuu​ggu​gca​aua​uga​ugu​cau) was cloned into pmirGLO vector. Similarly, the 3′UTR domain with mutated binding site of miR-489-3p (last 1,000 bp containing mutated miR-489-3p binding site: uuu​ggu​gca​aua​acu​aca​guu) was inserted into pmirGLO vector to construct SLC7A11-Mut. 293 T cells were seeded in 12-well plates with 1 × 10^5^ cells per well. The plasmids were transfected by lipofectamine 2000 under manufacturer’s instruction. After 24 h transfection, the cells were washed and lyzed, detected by using a dual luciferase reporter gene system (Promega, United States). The fluorescence density was detected by a microplate reader (PerkinElmer, United States).

### Western Blotting

HGC27 and SGC7901 cells at logarithmic growth phase were digested and placed in six-well plates at a density of 5 × 10^5^ cells per well, and transfected with miR-489-3p mimics, miR-489-3p inhibitor or NC. The cells were lyzed by RIPA lysis buffer (Sigma, China) added with the cocktail of protease inhibitors (Sigma). An equivalent amount of protein (30 μg) was separated in an SDS-PAGE and shifted onto the polyvinylidene fluoride (PVDF) membranes. The membranes were soaked in a fast-blocking reagent (Thermo) for 15 min, followed by incubation with primary antibodies against SLC7A11 (1: 1,000, Abcam, United States) and β-actin (1: 1,000, Abcam) at 4°C with gentle rocking all night. Next day, wash the membranes three times in TBST and incubate them in appropriate secondary antibodies (1: 1,000, Abcam). The membranes were visualized by a Enhanced chemiluminescence (ECL) reagent, and captured in a gel imaging system (BD Biosciences, United States).

### Statistical Analysis

Each experiment in this study was repeated at least three times. The results were shown as mean ± SD (standard deviation). Data analysis was performed by a Statistical Package for the Social Sciences (SPSS) 19.0 software. The *p* values were calculated using a student’s *t* test or one-way analysis of variance (ANOVA). *p* value less than 0.05 was set as the threshold for statical significance.

## Results

### Levobupivacaine Represses Growth of Gastric Cancer Cells

**FIGURE 1:**
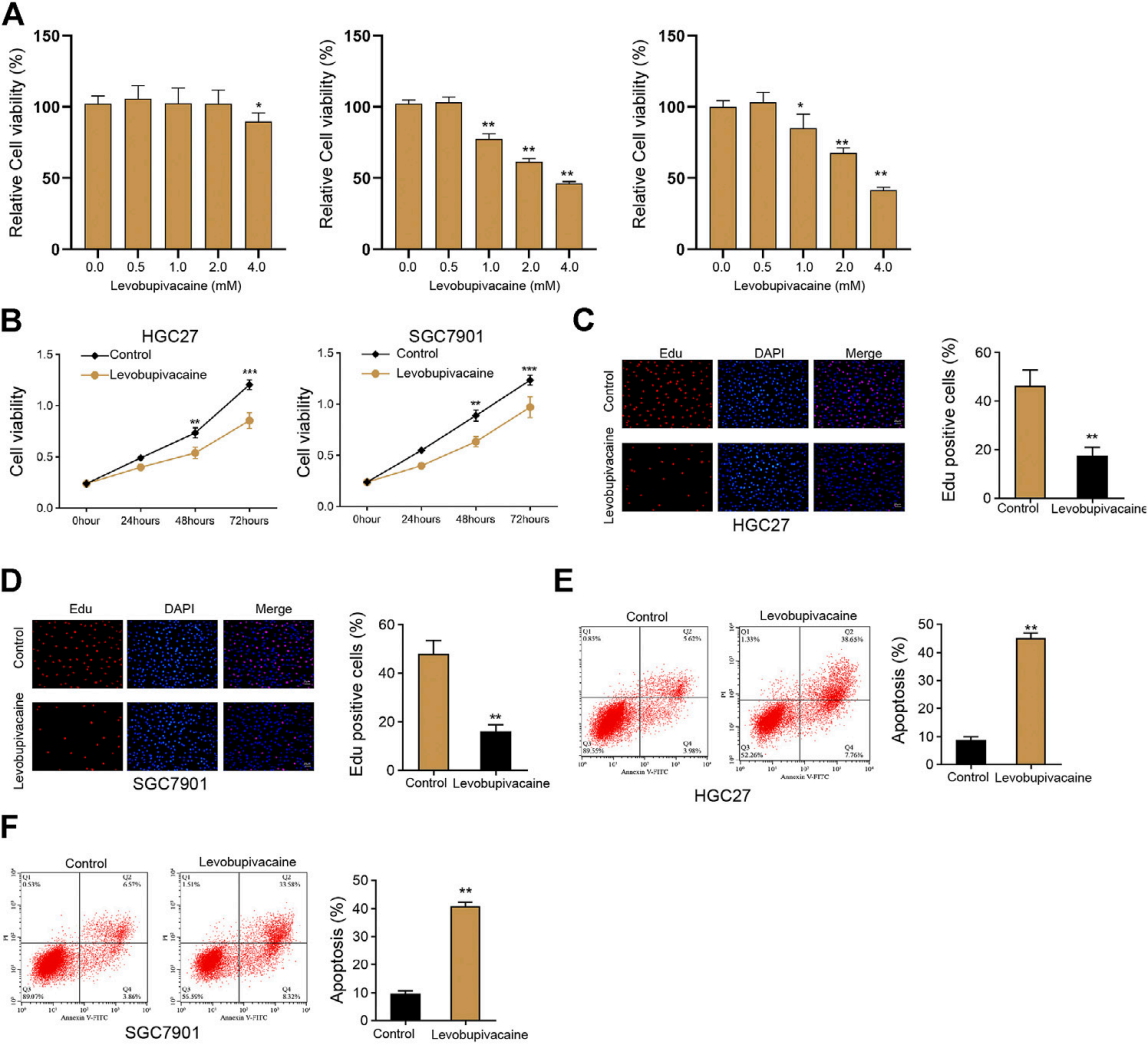
Levobupivacaine represses survival of gastric cancer cells *in vitro*. **(A)** The GES-1, HGC27, and SGC7901 cells were treated with levobupivacaine at the indicated concentrations. MTT assays for cell viability analysis. **(B–E)** HGC27 and SGC7901 cells were treated with saline or levobupivacaine (2 mM). **(B)** MTT assays for cell viability analysis. **(C,D)** Edu assays for cell proliferation analysis. **(E,F)** Flow cytometry analysis of apoptosis. The experiments were performed independently three times (mean ± SD, **p < 0.01).

We initially evaluated the effect of levobupivacaine on cell growth of gastric cancer cells *in vitro*. We found that levobupivacaine did not affected the viability of normal gastric epithelial GES-1 cell lines but inhibited the viability of HGC27 and SGC7901 cells in a dose-dependent manner (Figure 1A), and we selected the concentration of 2 mM in the subsequent analysis due to that 2 mM effectively repressed viability of HGC27 and SGC7901 cells and had no toxicity on GES-1 cells. We identified that the treatment of levobupivacaine suppressed HGC27 and SGC7901 cell viabilities (Figure 1B). Meanwhile, Edu assays showed that cell proliferation was attenuated by levobupivacaine in HGC27 and SGC7901 cells (Figures 1C,D). Moreover, levobupivacaine stimulated HGC27 and SGC7901 cell apoptosis *in vitro* (Figures 1E,F).

**FIGURE 2:**
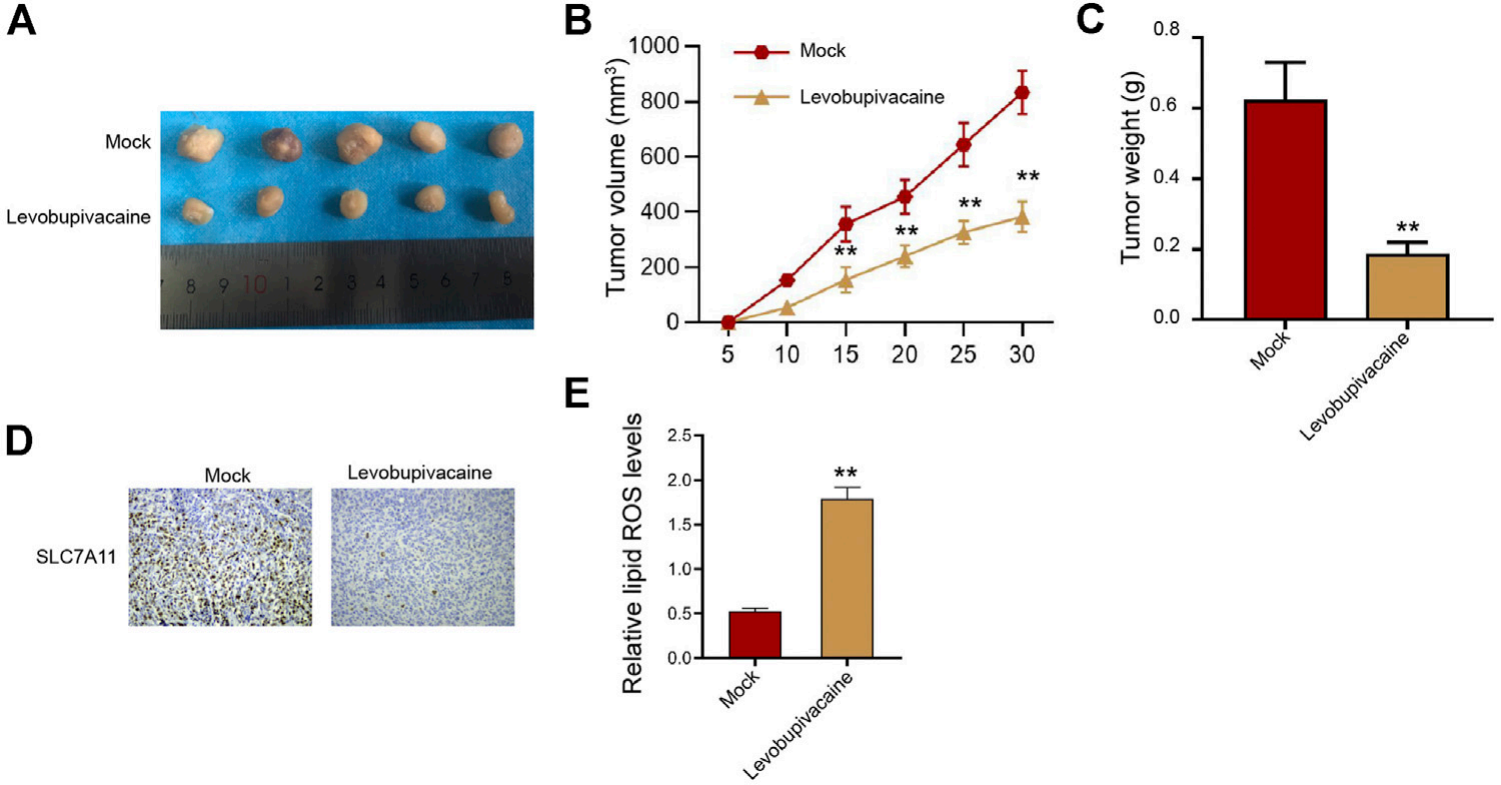
Levobupivacaine represses survival of gastric cancer cells *in vivo*. **(A–E)** The mice were injected with SGC7901 cells and treated with levobupivacaine (40 μmol/kg). The tumor tissues **(A)**, tumor volume **(B)**, and tumor weight. **(C)** were shown. *N* = 5, mean ± SD, **p < 0.01.

Furthermore, we assessed the impact of levobupivacaine on gastric cancer cell growth *in vivo*. We observed that SGC7901 cell growth was significantly inhibited by levobupivacaine in the nude mice (Figures 2A–C). Meanwhile, IHC analysis showed that the levels of SLC7A11 were repressed by levobupivacaine in the mice (Figure 2D). The lipid ROS accumulation was enhanced by levobupivacaine in the mice (Figure 2E).

### Levobupivacaine Enhances Ferroptosis of Gastric Cancer Cells

**FIGURE 3:**
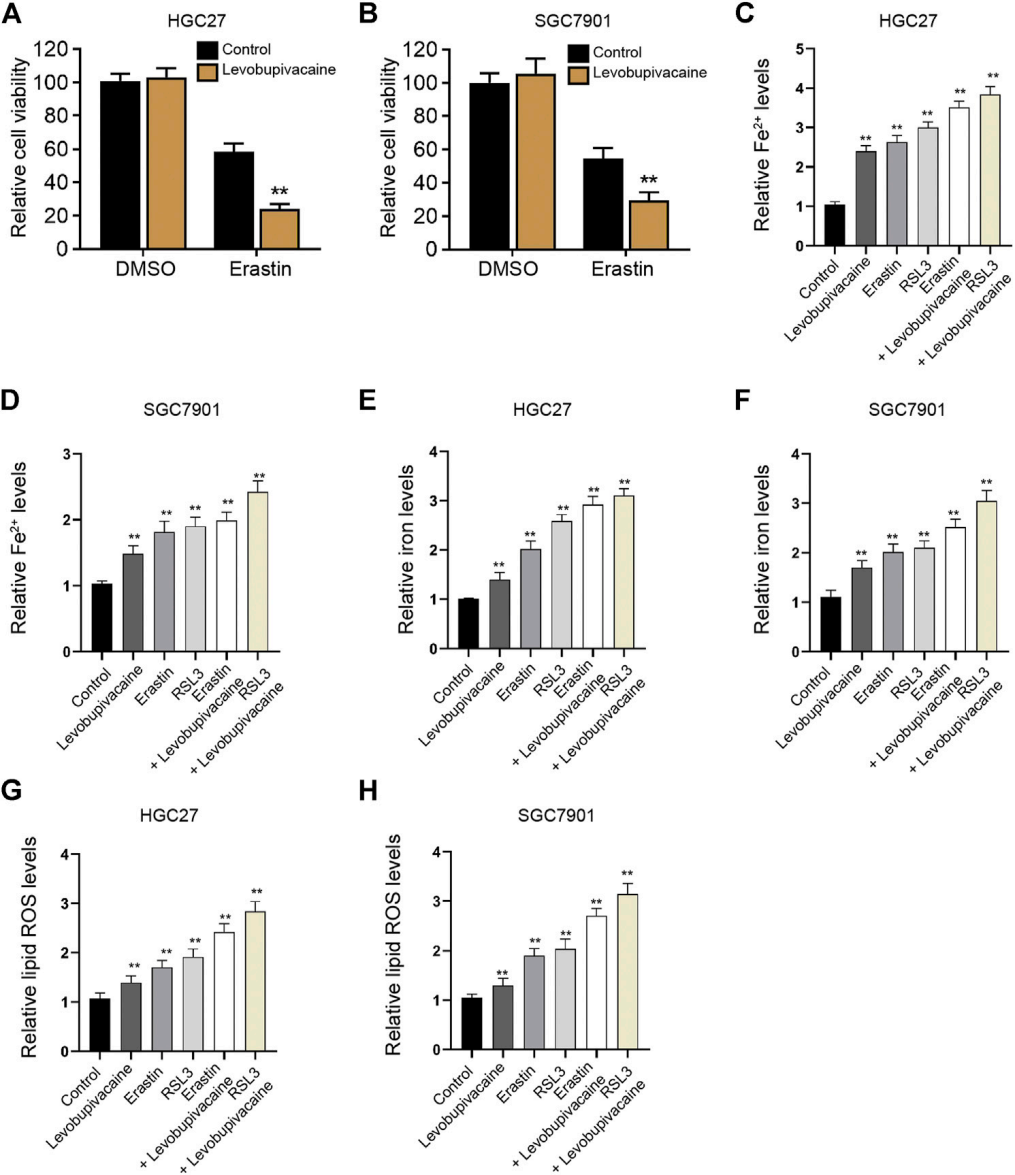
Levobupivacaine enhances ferroptosis of gastric cancer cells. **(A,B)** HGC27 and SGC7901 cells were co-treated with erastin (5 μM) and levobupivacaine (2 mM). MTT assays for cell viability analysis. **(C–H)** The erastin (5 μM) and RSL3 (1 μM)-stimulated HGC27 and SGC7901 cells were treated with saline or levobupivacaine (2 mM). The Fe^2+^ **(C,D)**, iron. **(E,F)**, and ROS levels **(G,H)** were analyzed. The experiments were performed independently three times (mean ± SD, **p < 0.01).

We then focused on the function of levobupivacaine in modulating ferroptosis in gastric cancer cells. We observed that the treatment of levobupivacaine enhanced erastin-induced inhibitory impact on HGC27 and SGC7901 cell viabilities (Figures 3A,B). The levels of Fe^2+^, iron, and lipid ROS were induced by the single treatment of levobupivacaine, erastin, and RSL3 in HGC27 and SGC7901 cells (Figures 3C–H), in which the combination treatment of levobupivacaine with erastin and RSL3 reforced the effect of erastin and RSL3 in the cells (Figures 3C–H), indicating that levobupivacaine enhances ferroptosis of gastric cancer cells.

### Levobupivacaine-Upregulated miR-489-3p Enhances Ferroptosis of Gastric Cancer Cells

**FIGURE 4:**
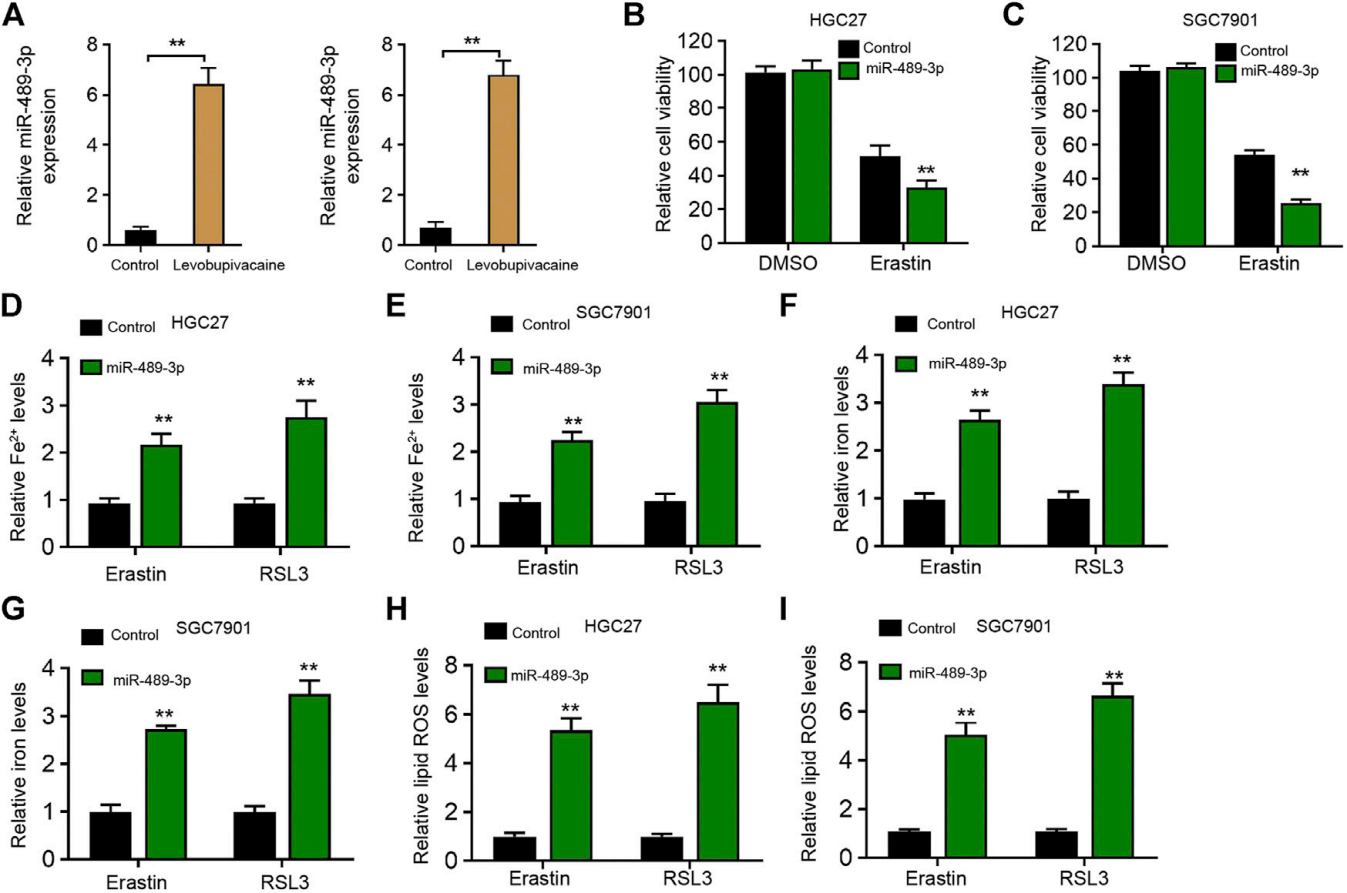
Levobupivacaine-upregulated miR-489-3p enhances ferroptosis of gastric cancer cells. **(A)** HGC27 and SGC7901 cells were treated with saline or levobupivacaine (2 mM). The qPCR analysis of miR-489-3p expression. **(B,C)** HGC27 and SGC7901 cells were co-treated with erastin (5 μM) and miR-489-3p mimic. MTT assays for cell viability analysis. **(D–I)** The erastin (5 μM) and RSL3 (1 μM)-stimulated HGC27 and SGC7901 cells were treated with miR-489-3p mimic. The Fe^2+^ **(D,E)**, iron **(F,G)**, and ROS levels **(H,I)** were analyzed. The experiments were performed independently three times (mean ± SD, **p < 0.01).

Next, we identified that levobupivacaine was able to enhance the expression of miR-489-3p in HGC27 and SGC7901 cells (Figure 4A). Moreover, miR-489-3p mimic promoted erastin-induced inhibitory influence on HGC27 and SGC7901 cell viabilities (Figures 4B,C). The levels of Fe^2+^ and iron were increased by miR-489-3p mimic in erastin and RSL3-stimulated HGC27 and SGC7901 cells (Figures 4D–G). Meanwhile, the treatment of miR-489-3p mimic induced lipid ROS accumulation in erastin and RSL3-stimulated HGC27 and SGC7901 cells (Figures 4H,I).

### MiR-489-3p is Involved in Levobupivacaine-Induced Ferroptosis of Gastric Cancer Cells

**FIGURE 5:**
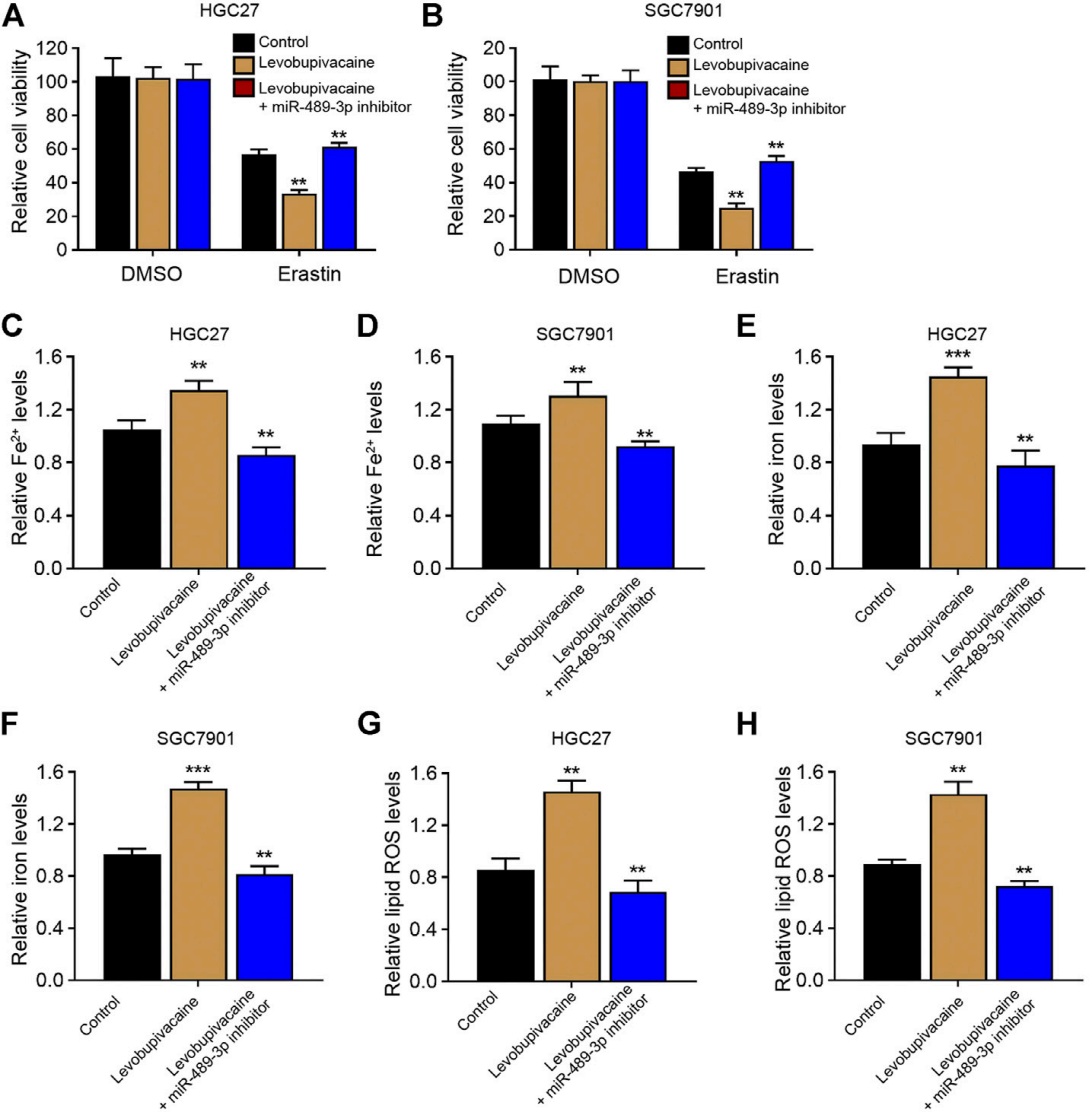
MiR-489-3p is involved in levobupivacaine-induced ferroptosis of gastric cancer cells. **(A–H)** The erastin (5 μM)-treated HGC27 and SGC7901 cells were co-treated with levobupivacaine and miR-489-3p inhibitor. **(A,B)** MTT assays for cell viability analysis. The Fe^2+^ **(C,D)**, iron **(E,F)**, and ROS levels **(G,H)** were analyzed. The experiments were performed independently three times (mean ± SD, **p < 0.01).

We then analyzed whether miR-489-3p was involved in levobupivacaine-induced ferroptosis of gastric cancer cells. We found that levobupivacaine reduced cell viabilities of erastin-treated HGC27 and SGC7901 cells, while miR-489-3p inhibitor rescued the viabilities (Figures 5A,B). Meanwhile, the levels of Fe^2+^, iron, and lipid ROS were increased by the treatment of levobupivacaine in erastin-treated HGC27 and SGC7901 cells, in which the inhibition of miR-489-3p reversed these results (Figures 5C–H).

### MiR-489-3p Targets Ferroptosis Inhibitor SLC7A11 in Gastric Cancer Cells

**FIGURE 6:**
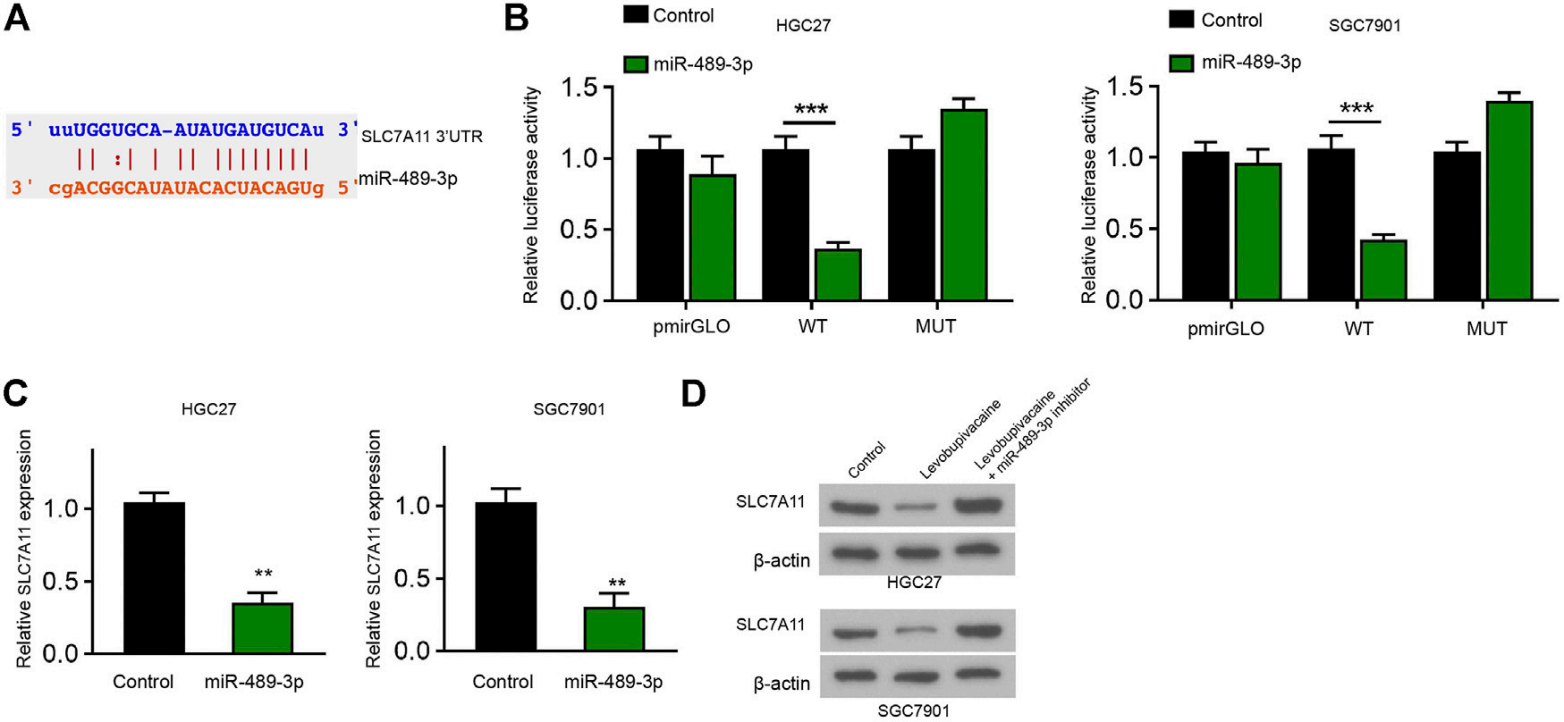
MiR-489-3p targets ferroptosis inhibitor SLC7A11 in gastric cancer cells. **(A)** The binding prediction between SLC7A11 and miR-489-3p in ENCORI database. **(B,C)** HGC27 and SGC7901 cells were treated with miR-489-3p mimic. The luciferase activity of SLC7A11 mRNA 3′UTR **(B)** and SLC7A11 mRNA expression **(D)** were detected **(D)** HGC27 and SGC7901 cells were co-treated with levobupivacaine and miR-489-3p inhibitor. Western blot analysis of SLC7A11 expression. The experiments were performed independently three times (mean ± SD, **p < 0.01, ***p < 0.001).

We identified the binding site between SLC7A11 and miR-489-3p in the Encyclopedia of RNA Interactomes (ENCORI) database (Figure 6A). Meanwhile, the treatment of miR-489-3p mimic reduced luciferase activity of SLC7A11 mRNA 3′UTR in HGC27 and SGC7901 cells (Figure 6B). The mRNA levels of SLC7A11 were decreased by miR-489-3p in HGC27 and SGC7901 cells (Figure 6C). Meanwhile, levobupivacaine repressed SLC7A11 expression and miR-489-3p inhibitor could reverse this effect in HGC27 and SGC7901 cells (Figure 6D).

### SLC7A11 is Involved in miR-489-3p-Induced Ferroptosis of Gastric Cancer Cells

**FIGURE 7:**
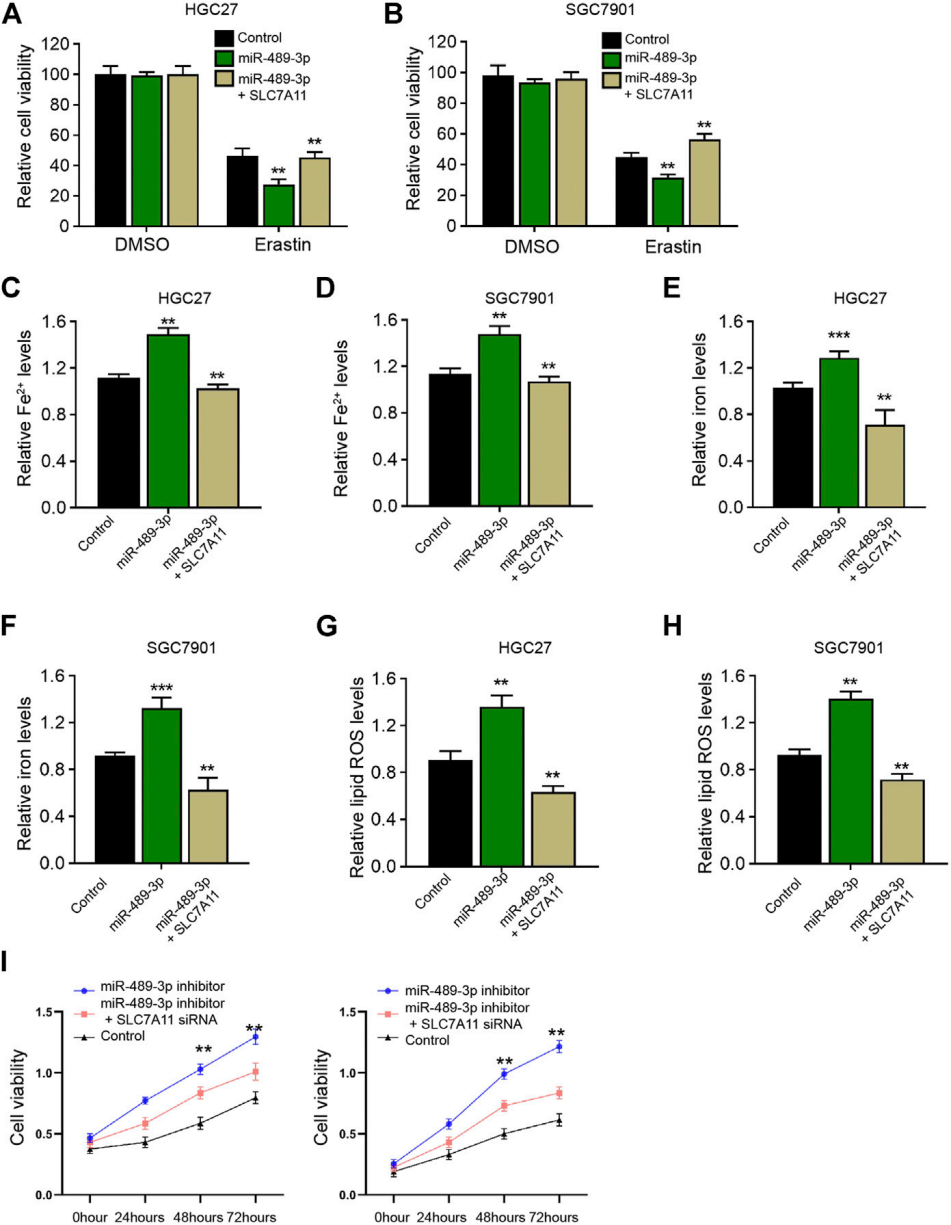
SLC7A11 is involved in miR-489-3p-induced ferroptosis of gastric cancer cells. **(A–H)** The erastin (5 μM)-treated HGC27 and SGC7901 cells were co-treated with miR-489-3p mimic and SLC7A11 overexpression vectors. **(A,B)** MTT assays for cell viability analysis. The Fe^2+^ **(C,D)**, iron **(E,F)**, and ROS levels **(G,H)** were analyzed **(I)**. The erastin (5 μM)-treated HGC27 and SGC7901 cells co-treated with miR-489-3p inhibitor and SLC7A11 siRNA. The cell viability was detected by MTT assays. The experiments were performed independently three times (mean ± SD, **p < 0.01).

We then detected whether SLC7A11 was involved in miR-489-3p-enhanced ferroptosis of gastric cancer cells. We observed that miR-489-3p inhibited cell viabilities of erastin-treated HGC27 and SGC7901 cells, while SLC7A11 overexpression rescued the viabilities (Figures 7A,B). Meanwhile, the levels of Fe^2+^, iron, and lipid ROS were induced by the treatment of miR-489-3p mimic in erastin-treated HGC27 and SGC7901 cells, in which the overexpression of SLC7A11 reversed this effect (Figures 7C–H). Moreover, the suppression of miR-489-3p by miR-489-3p inhibitor enhanced the viabilities of erastin-treated HGC27 and SGC7901 cells, while the depletion of SGC7901 by siRNA could reversed this effect (Figure 7I).

### Levobupivacaine/miR-489-3p/SLC7A11 Axis Attenuates Gastric Cancer Cell Proliferation *in vitro*

**FIGURE 8:**
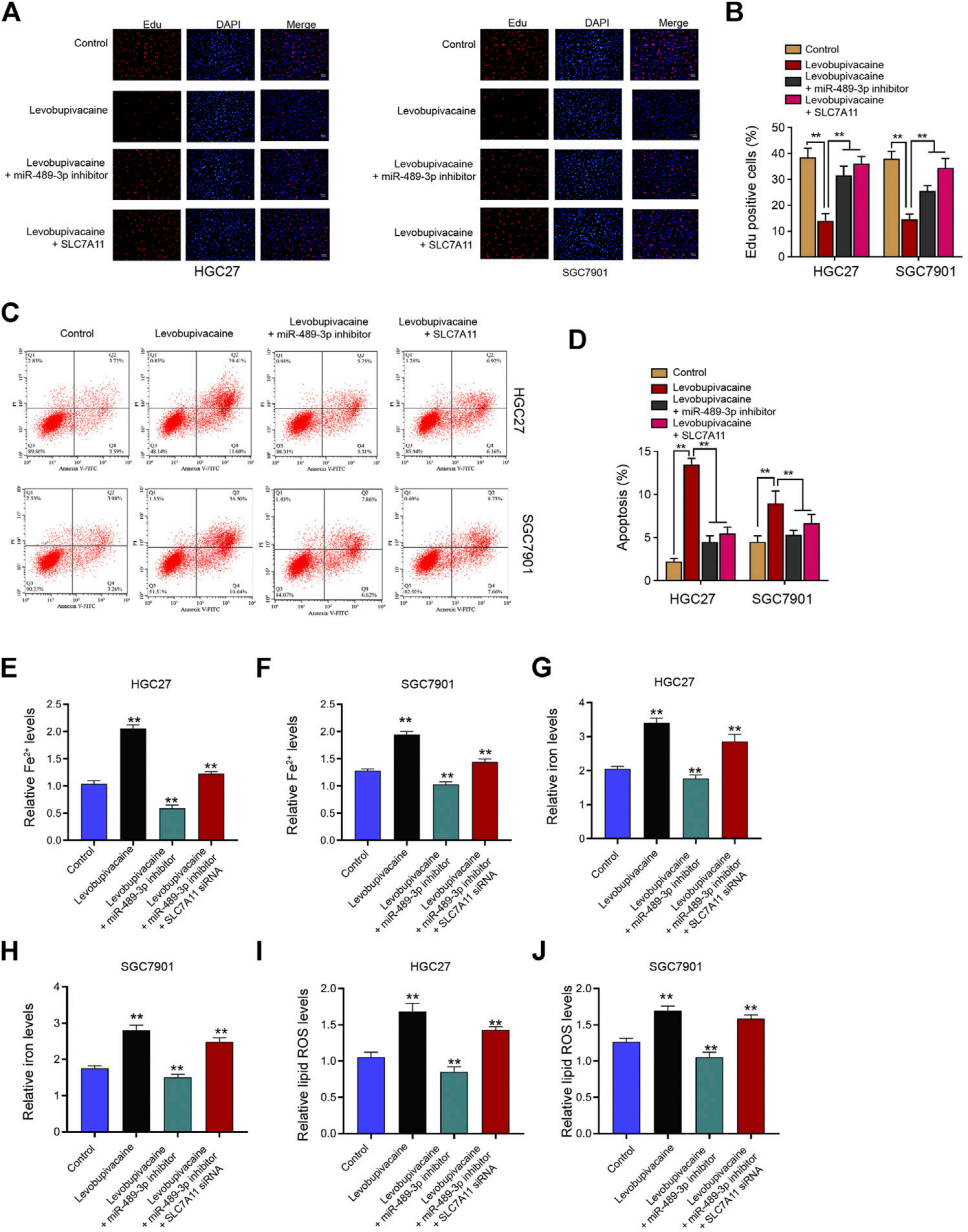
Levobupivacaine/miR-489-3p/SLC7A11 axis attenuates gastric cancer cell proliferation *in vitro*. **(A–D)** HGC27 and SGC7901 cells were co-treated with levobupivacaine and SLC7A11 overexpression vectors or miR-489-3p inhibitor. **(A,B)** MTT assays for cell viability analysis. **(C,D)** Flow cytometry analysis of apoptosis. **(E–J)** HGC27 and SGC7901 cells were co-treated with levobupivacaine and miR-489-3p inhibitor or co-treated with levobupivacaine, miR-489-3p inhibitor, and SLC7A11 siRNA. The Fe^2+^ **(E,F)**, iron **(G,H)**, and ROS levels **(I,J)** were analyzed. The experiments were performed independently three times (mean ± SD, **p < 0.01).

Next, we further evaluated the impact of levobupivacaine/miR-489-3p/SLC7A11 axis on gastric cancer progression *in vitro*. Our data revealed that the treatment of levobupivacaine repressed Edu-positive HGC27 and SGC7901 cells and miR-489-3p inhibitor or SLC7A11 overexpression was able to rescue this phenotype (Figures 8A,B). Meanwhile, the HGC27 and SGC7901 cell apoptosis was induced by levobupivacaine, in which SLC7A11 overexpression or miR-489-3p inhibition reversed the effect (Figures 8C,D). Moreover, the levels of Fe^2+^, iron, and lipid ROS were induced by levobupivacaine and miR-489-3p inhibition could attenuate the induction in HGC27 and SGC7901 cells, in which the depletion of SLC7A11 futher reversed the effect of miR-489-3p inhibition (Figures 8E–J).

**FIGURE 9:**
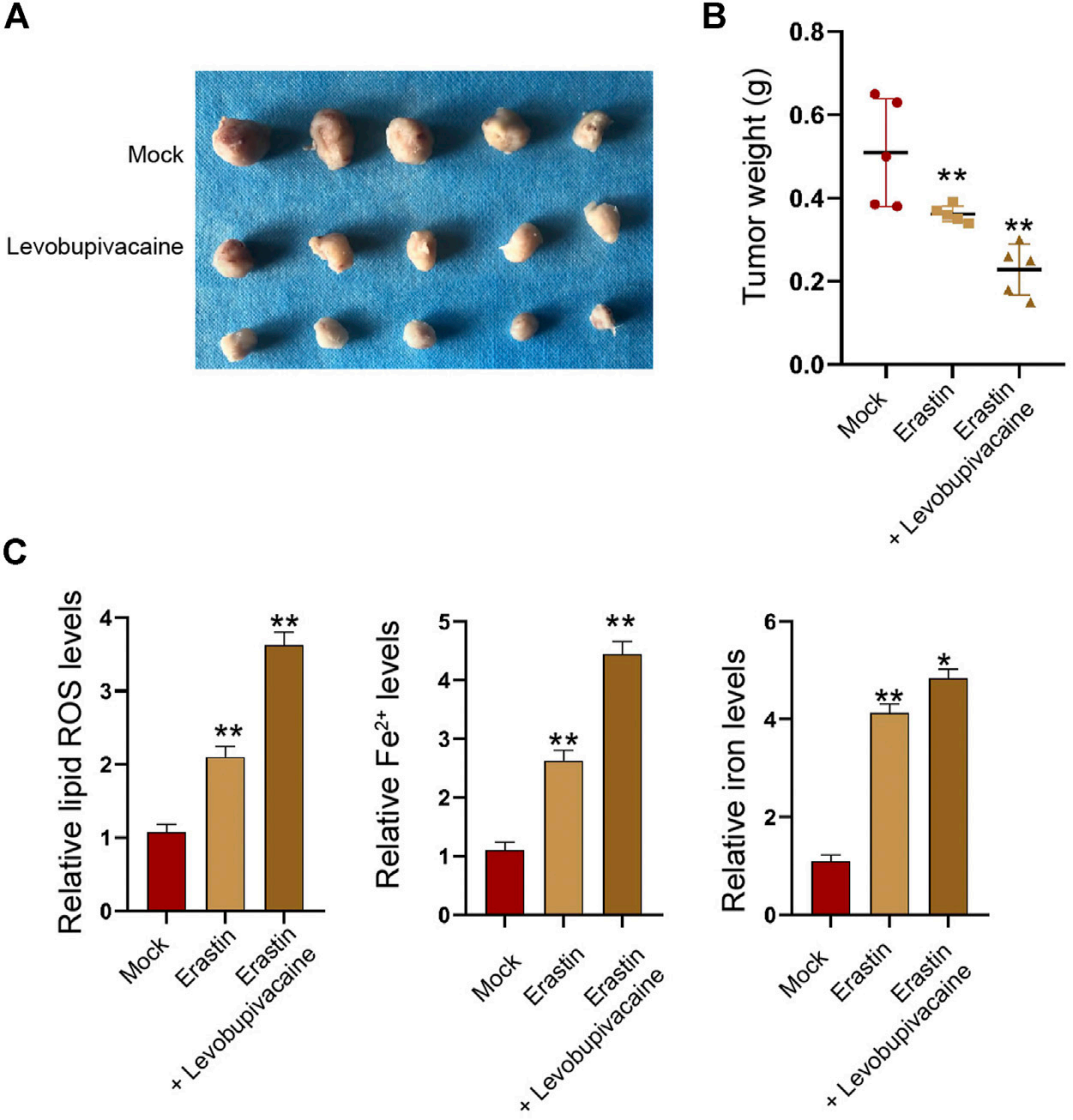
Levobupivacaine enhances inhibitory effect of erastin on gastric cancer cell growth *in vivo*. The mice were injected with SGC7901 cells and treated with erastin (15 mg/kg) and levobupivacaine (40 μmol/kg). The tumor tissues **(A)** and tumor weight **(B)** were shown. **(C)** The lipid ROS, Fe^2+^, iron levels were analyzed. *N* = 5, mean ± SD, **p < 0.01.

Moreover, tumorigenicity analysis identified that the treatment of erastin repressed the SGC7901 cell growth and enhanced Fe^2+^, iron, and lipid ROS levels in the nude mice (Figures 9A–C), and the co-treatment of levobupivacaine with erastin could reinforce the effect of erastin (Figures 9A–C).

## Discussion

Gastric cancer is one of the most the prevalent cancers and the treatment strategies for patients with gastric cancer are limited. The local anesthetic levobupivacaine presents potential anti-cancer properties in several cancers, but its correlation with gastric cancer and ferroptosis is poor understood. We identified the novel function of levobupivacaine in regulating ferroptosis of gastric cancer cells.

Previous studies show that ferroptosis is an essential process in gastric cancer development. MiR-522 contributes to chemoresistance and represses ferroptosis of gastric cancer cells (Zhang et al., 2020). Tanshinone IIA promotes ferroptosis by p53-regulated downregulation of SLC7A1 in gastric cancer (Guan et al., 2020). The abnormal regulation of lipid metabolism and ferroptosis are involved in gastric cancer progression (Sun et al., 2020). Here, we identified that levobupivacaine induced ferroptosis of gastric cancer cells. It demonstrates a new function of levobupivacaine in modulating ferroptosis of gastric cancer cells. Meanwhile, the clinical value of levobupivacaine targeting ferroptosis in gastric cancer is needed to explore. It has been reported levobupivacaine has multiple anti-cancer activities. Levobupivacaine represses cell survival by inhibiting the Akt/mTOR signaling in breast cancer cells (Kwakye et al., 2020). Levobupivacaine attenuates migration and proliferation of melanoma and breast cancer cells (Castelli et al., 2020). Levobupivacaine induces an inhibitory effect on the growth of colon cancer cells (Li et al., 2019). Our data showed that levobupivacaine repressed gastric cancer cell growth *in vitro* and *in vivo*. Due to the limitation of application concentration of levobupivacaine, the drug-combined strategy with levobupivacaine may benefit the clinical treatment of gastric cancer, which is required further investigation. Meanwhile, we presented a similar data in the HGC27 and SGC7901 cells, and we will use other gastric cancer cell lines to prove our findings in future investigation.

SLC7A11 plays a critical function in modulation of ferroptosis and cancer development. Moreover, it has been reported that GDF15 inhibition contributes to erastin-stimulated ferroptosis by inhibiting SLC7A11 (Chen et al., 2020). KDM4A-regulated SLC7A11 decreases ferroptosis of osteosarcoma cells (Chen et al., 2021). Circular RNA circEPSTI1 represses the development of cervical cancer by targeting SLC7A11 (Wu et al., 2021). Meanwhile, miR-489-3p has been identified as a tumor suppressor. MiR-489-3p represses cell migration and proliferation by downregulating histone deacetylase two in bladder cancer cells (Sun et al., 2020). MiR-489 inhibits invasion and proliferation of bladder cancer (Li et al., 2016). The inhibition of miR-489-3p contributes to the metastasis of osteosarcoma *via* regulating PAX3/MET signaling (Liu et al., 2017). However, the correlation of SLC7A11 with miR-489-3p in regulating cancer development is still unclear. Our data showed that miR-489-3p upregulated by levobupivacaine contributed to ferroptosis of gastric cancer cells by targeting SLC7A11. It indicates an innovative mechanism of levobupivacaine/miR-489-3p/SLC7A11 axis in the regulation of gastric cancer. Moreover, it has been reported that miR-489-3p suppresses cell proliferation, migration, invasion, and glycolysis in cancer cells, there is little known on ferroptosis. We reported the new function of miR-489-3p in regulating ferroptosis of gastric cancer cells. MiR-489-3p/SLC7A11 axis may just one of the downstream mechanisms underlying levobupivacaine-induced ferroptosis and anti-cancer activities and more related studies are needed to perform in the future. It has been reported that levobupivacaine inhibits cell survival by suppressing the Akt/mTOR signaling in breast cancer cells (Kwakye et al., 2020). The correlation of Akt/mTOR with levobupivacaine in modulating ferroptosis of gastric cancer cells needs to verify in future investigations.

Accordingly, we concluded that the local anesthetic levobupivacaine induced ferroptosis of gastric cancer cells to repress gastric cancer cell growth by miR-489-3p/SLC7A11 axis. Levobupivacaine may be applied as an anti-cancer agent in gastric cancer, especially in the combination treatment with other anti-cancer drugs.

## Data Availability

The original contributions presented in the study are included in the article/Supplementary Material, further inquiries can be directed to the corresponding author.
